# The burden of diabetes-associated multiple long-term conditions on years of life spent and lost

**DOI:** 10.1038/s41591-024-03123-2

**Published:** 2024-08-01

**Authors:** Edward W. Gregg, Adrian Pratt, Alex Owens, Emma Barron, Rupert Dunbar-Rees, Eirion T. Slade, Nasrin Hafezparast, Chirag Bakhai, Paul Chappell, Victoria Cornelius, Desmond G. Johnston, Jacqueline Mathews, Jason Pickles, Ellie Bragan Turner, Gary Wainman, Kate Roberts, Kamlesh Khunti, Jonathan Valabhji

**Affiliations:** 1 RCSI University of Medicine and Health Sciences, Dublin, Ireland; 2 School of Public Health, Imperial College London, London, UK; 3 NHS Arden & GEM Commissioning Support Unit, Leicester, UK; 4 NHS England, London, UK; 5 Chelsea and Westminster Hospital NHS Foundation Trust, London, UK; 6 Department of Metabolism, Digestion and Reproduction, Faculty of Medicine, Imperial College London, London, UK; 7 Outcomes Based Healthcare Ltd, London, UK; 8 Bedfordshire, Luton and Milton Keynes Integrated Care Board, Luton, UK; 9 Department of Diabetes & Endocrinology, St Mary’s Hospital, Imperial College Healthcare NHS Trust, London, UK; 10 National Institute for Health and Care Research Clinical Research Network National Coordination Centre, Faculty of Medicine and Health, University of Leeds, Leeds, UK; 11 Diabetes Research Centre, University of Leicester, Leicester, UK

**Keywords:** Diabetes, Diabetes complications, Metabolic disorders

## Abstract

Diabetes mellitus is a central driver of multiple long-term conditions (MLTCs), but population-based studies have not clearly characterized the burden across the life course. We estimated the age of onset, years of life spent and loss associated with diabetes-related MLTCs among 46 million English adults. We found that morbidity patterns extend beyond classic diabetes complications and accelerate the onset of severe MLTCs by 20 years earlier in life in women and 15 years earlier in men. By the age of 50 years, one-third of those with diabetes have at least three conditions, spend >20 years with them and die 11 years earlier than the general population. Each additional condition at the age of 50 years is associated with four fewer years of life. Hypertension, depression, cancer and coronary heart disease contribute heavily to MLTCs in older age and create the greatest community-level burden on years spent (813 to 3,908 years per 1,000 individuals) and lost (900 to 1,417 years per 1,000 individuals). However, in younger adulthood, depression, severe mental illness, learning disabilities, alcohol dependence and asthma have larger roles, and when they occur, all except alcohol dependence were associated with long periods of life spent (11–14 years) and all except asthma associated with many years of life lost (11–15 years). These findings provide a baseline for population monitoring and underscore the need to prioritize effective prevention and management approaches.

## Abstract (web summary)

In a national study of 46 million adults, living with diabetes-related multiple long-term health conditions(MLTCS), particularly during young adulthood, led to extended periods of time with MLTCS, while also substantially reducing the years of life.

## Main

Type 2 diabetes is a major conduit for diverse forms of morbidity because of the systemic effects of chronic hyperglycemia, insulin resistance, and accompanying endothelial, inflammatory and other pathophysiological dysfunctions^1^. This has been observed most in its strong association with a cadre of microvascular and macrovascular complications, including diseases of the cardiovascular system, eye, foot and kidneys^2,3^. These complications have been the target of comprehensive guideline-driven prevention efforts and risk factor management and have been accompanied by overall reductions in rates of long-term complications, particularly cardiovascular complications, in many high-income countries^3,4^.

Diabetes is also associated with effects on morbidity far beyond the classic complications, as highlighted by previous studies associating diabetes with hospitalizations and deaths due to cancer, infections, respiratory disease, liver disease and dementia^5–7^. Although these latter conditions are less specific in their association with diabetes than classic microvascular complications, they still carry moderately high relative risks, and importantly, their rates are either stagnant or have been increasing over time. Whether due to increasing life expectancy or increases in the etiological drivers of these conditions, such as obesity, they appear to be representing a diversification of diabetes-related complications and contributing to a high burden of multiple long-term conditions (MLTCs; or multimorbidity), wherein, compared to prior cohorts, adults with diabetes live longer but may have more comorbid conditions despite experiencing a decrease in the incidence of cardiovascular disease (CVD)^7–9^.

The diversification of complications could be due to a combination of factors, with different drivers at each end of the age spectrum—increasing life expectancy and reduced CVD risk at the older end of the age distribution, and increasing obesity, changing risk profiles and early-onset diabetes at the young end of the age distribution^10–12^. Thus, the burden of complications may be shifting toward younger adulthood and shifting the balance from CVD to non-CVD complications, while driving a growing crisis of MLTCs^13,14^. MLTCs have therefore emerged as an important priority for health systems, including the National Health Service (NHS) in England, because of the burden on clinical and self-management, health systems, costs and quality of life if increasing segments of the population spend long periods of life with multiple conditions^14^. The fragmented and single-disease model of care in many health systems makes for further challenges in adapting to complex patient needs with MLTCs. The apparent growth in MLTCs in England may have been partially fueled by the multidecade growth in type 2 diabetes prevalence, and conversely, MLTCs may represent the next major transition and challenge in the type 2 diabetes epidemic^5,15^.

Unfortunately, attempts to characterize and monitor the burden of MLTCs among people with diabetes and other conduit conditions have been limited by the presence of relatively crude metrics. For example, people with diabetes are more likely to develop two, three, four or more conditions^16–18^, but these estimates obscure the great diversity in types and severity of the impact of MLTCs^19^. Clustering analyses have also suggested that in addition to the common microvascular and macrovascular pathways, comorbid conditions may aggregate around particular mental health outcomes or, separately, aging-related conditions^16,18,20^. These findings provide insights into epidemiology but have not clearly characterized the MLTC phenotypes that are driving the contemporary burden and impact on life expectancy through the life course. Similarly, it is unclear whether the MLTC burden is driven by combinations of conditions that are ‘concordant’ (that is, comprised primarily of etiologically linked classic complications) or increasingly comprised of a ‘discordant’ mix of emerging, nonspecific complications^21^.

One of the biggest gaps in the epidemiology of diabetes-related MLTCs is the lack of quantification of years spent and years of life reduced associated with MLTCs, in part due to the inherent difficulty in computation. Incorporating these metrics is essential for policymakers, researchers and people with MLTCs to understand the burden on health systems and individuals across the life course^22^. Providing time-based, precise metrics could facilitate a better understanding of modifiable risk factors of MLTCs and inform the health system's response and models of care and prevention for the challenge of MLTCs.

In the present analyses, we examine the burden of MLTCs among 46.7 million adults in England, using the National Bridges to Health Segmentation Dataset^23^. The segmentation dataset assembles data from 15 separate sources and includes all adults registered with a general practice (GP) in England. Previous work has suggested that over 98% of the English population are registered with a GP, so the data are highly representative of the English population^23–25^. By using information on the longitudinal onset of new conditions on 35 conditions, we developed a series of new metrics to assess the MLTC burden associated with diabetes in England^26,27^. In addition to describing the traditional metrics of the prevalence of counts of common conditions in the English population, we estimate the age of onset and years spent and lost due to MLTCs among the population with diabetes and express this burden from the perspective of both individuals and communities. These analyses are intended to provide a basis for monitoring the national burden of MLTCs, to support better health service resource allocation and commissioning decisions and to provide the infrastructure to assess impact of new initiatives in prevention and care.

## Results

### Diabetes-related MLTC prevalence

**Fig. 1:**
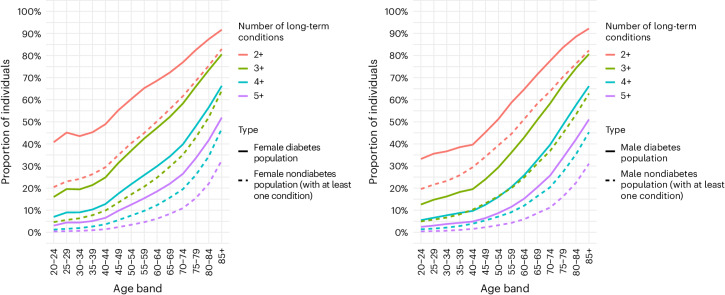
Prevalence of MLTCs among women and men with and without diabetes, by age. Among persons with diabetes, a number of conditions include diabetes. The comparative population without diabetes has at least one index condition at any time (except for diabetes).

Of the 46,748,714 adults who were registered with a GP in England and alive on 31 March 2020, 3,663,429 (7.8%) had diagnosed diabetes, including type 1, type 2 and other forms of diabetes. Across all age ranges, adults with diabetes were more likely to have MLTC than the population with one of the other 34 comorbid conditions (Fig. 1). By the age of 50 years, about one-third of adults with diabetes (37.0% women and 29.4% men) have at least three conditions, compared to 17.2% women and 16.4% men in the general population without diabetes. The prevalence of three conditions does not reach this level in the general population until after the age of 70 years in women and the age of 65 years in men. The differences in prevalence between persons with and without diabetes were greater at younger ages and for more severe levels of MLTCs. For example, women aged 40–44 years with diabetes were more than three times as likely as those without diabetes (12.8% versus 3.6% prevalence) and men more than twice as likely as those without diabetes (9.7% versus 3.9% prevalence) to have four conditions or more. By the age of 70 years, 40% of women and men with diabetes had at least four conditions, compared to 20% among those without diabetes.

### MLTC patterns by life stages

**Fig. 2:**
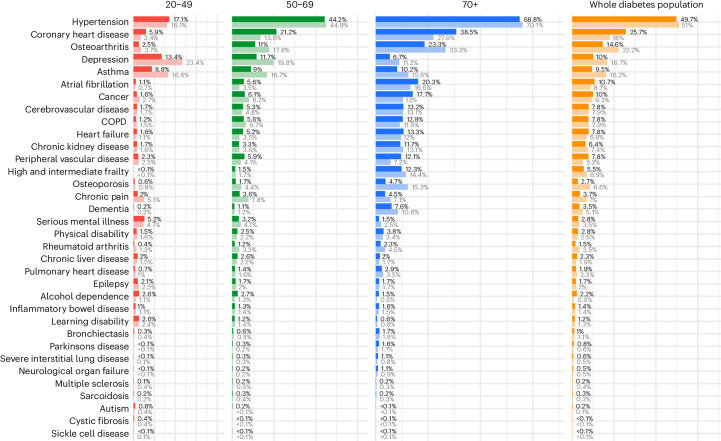
Prevalence of comorbid conditions among adults aged ≥18 years with diabetes, by sex and age group. Men are represented by dark bars and women are represented by light bars.

The comorbid conditions associated with diabetes are diverse, with a high prevalence of hypertension (51% of women and 50% of men), coronary heart disease (CHD; 25.7% men and 18% women) and osteoarthritis (14.6% men and 22.2% women), followed by depression (10.0% men and 16.7% women) and asthma (9.5% men and 16.2% women). The prevalence of CHD is 8% points higher in men than in women, whereas osteoarthritis, depression and asthma are each 6–7% points more common in women than in men (Fig. 2). A second tier of conditions, including atrial fibrillation, cancer, cerebrovascular disease, chronic obstructive pulmonary disease (COPD), heart failure, chronic kidney disease (CKD), peripheral vascular disease (PVD), frailty, osteoporosis and chronic pain, ranged in prevalence from 5% to 10%.

Age-stratified analyses reveal variations in the ranking of the prevalence of diabetes-associated comorbid conditions (Fig. 2). Older adults (age ≥ 70 years) have a higher prevalence of hypertension (68.8% men and 70.1% women), CHD (38.5% men and 27.6% women), osteoarthritis (23.3% men and 33.3% women) and atrial fibrillation (20.3% men and 16.6% women), whereas cancer, cerebrovascular disease, heart failure and CKD ranged from 12% to 20% in prevalence. Middle-aged adults had similar contributors to MLTCs but with roughly 20–50% lower absolute prevalence

For younger adults (age = 20–49 years), hypertension is also one of the most prevalent conditions (17.1% men and 16.1% women) and CHD is already present in 5.9% of men. However, apart from hypertension and CHD, younger adults do not share the pattern or ranking of morbidity seen in older adults. Instead, the more common chronic conditions are depression, with prevalence at 18% (13.4% men and 23.4% women), asthma at 13% (8.8% men and 16.6% women) and serious mental illness at 5% (5.2% men and 4.7% women).

**Extended Data Fig. 1:**
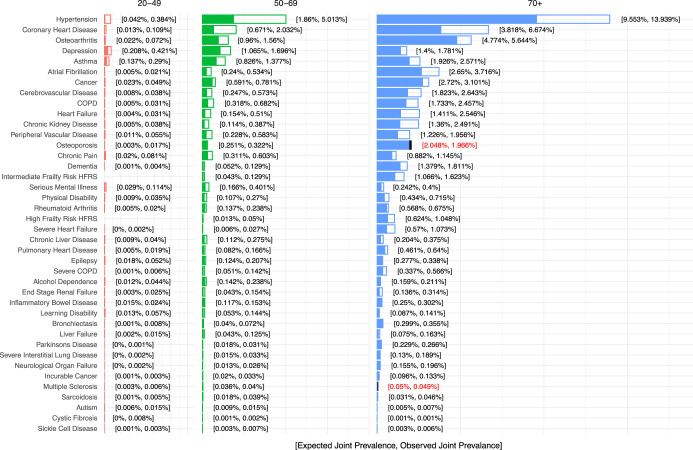
Observed and expected prevalence of having both diabetes and comorbid conditions among overall adults population aged > 20 years. Full bar represents the observed joint prevalence of diabetes with each condition; blue segment of the bar is the joint prevalence that would be expected with no etiologic association among the two conditions.

When expressed as the absolute prevalence of all diabetes-related bivariate combinations, the number of excess cases exceeded the numbers that would be expected based on chance alone for most conditions (Extended Data Fig. 1). For example, of the 14% of adults with both diabetes and hypertension, 4.5% are beyond what was expected by chance. Among younger strata (age = 20–49 years), the prevalence is lower (5% for the age of 65–69 years and 0.4% for the age of 20–49 years), but the majority of cases are greater than expected by chance.

### Years spent with and lost to MLTCs

**Fig. 3:**
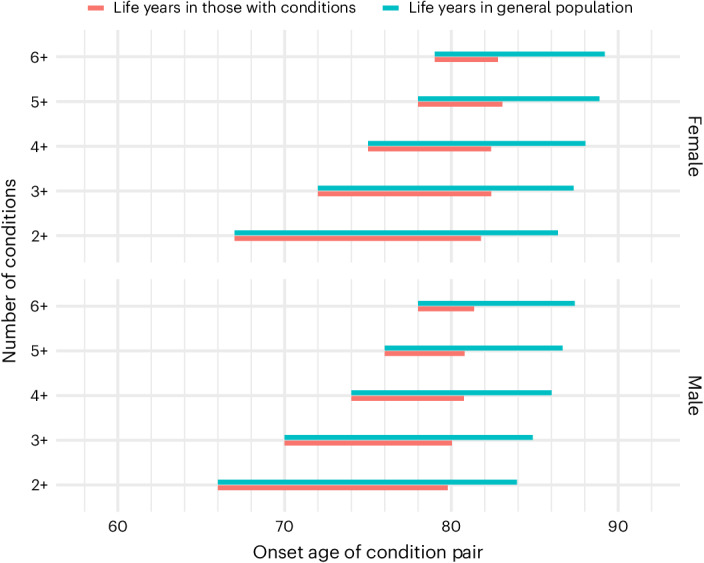
Median age of onset and years of life spent and lost associated with the number of conditions (including diabetes) among women and men in England. Segment in red represents period from median age of onset of condition and death among persons who develop the noted number of conditions. Segment in blue represents the period of expected life from the equivalent age among the general population with and without comorbid conditions.

The median age of onset of MLTCs for at least two conditions was age 67 years in women and 66 years in men, whereas persons who developed three, four, five or six conditions had median onsets of their MLTC combinations in their 70s (Fig. 3). Women consistently had 1–2 years later onset of MLTC, but when it occurred, they lost roughly the same number of years of life as men relative to their general population peers. Persons with more conditions had fewer years living with MLTCs and died earlier compared to the general population without MLTCs; for example, persons with three conditions live about 10 years with the MLTCs after entry into that group and lose five years relative to the general population, whereas those with at least five conditions live 5 years and lose 6 years (Fig. 3).

**Fig. 4:**
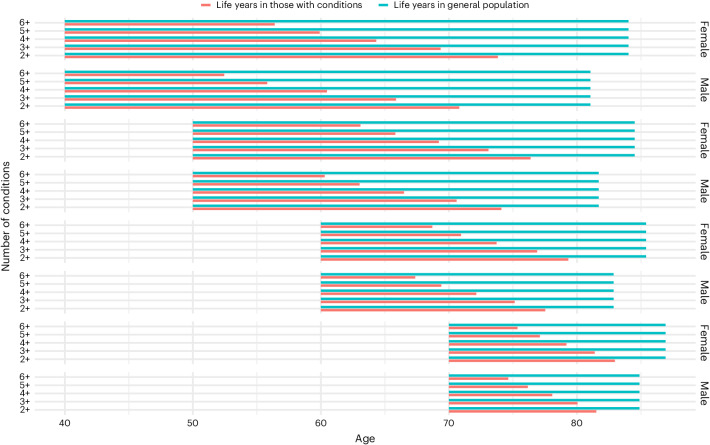
Median number of expected years of life spent before death associated with the number of prevalent MLTCs, according to age, among women and men in England. Segment in red represents years before death among persons with diabetes, according to age and number of conditions. Segment in blue represents the age-conditional life expectancy among the general same-age population with and without MLTCs.

When MLTCs occur in younger adulthood, the impact on life years spent and lost is more substantial (Fig. 4). Among persons with prevalent MLTCs at the age of 40 years, each additional condition is associated with about four fewer years of life compared to the general population with and without conditions (Fig. 4). By the age of 60 years, each additional condition was associated with about two fewer years of life spent with conditions. A 40-year-old with diabetes with three conditions loses about 14 years of life, whereas a 60-year-old with three conditions total loses about 8 years of life. Women spent about four more years with MLTCs than men from age of 40 years. In older ages, such as the age of 70 years, fewer years are spent and fewer years are lost to a given level of MLTCs relative to younger ages.

**Fig. 5:**
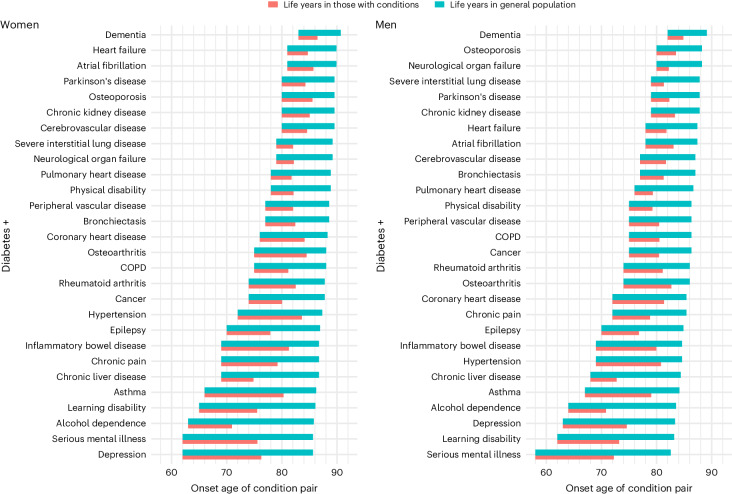
Median age of onset and years of life spent and lost associated with the combination of diabetes and additional comorbid conditions among women and men in England. Segment in red represents period from median age of onset of condition and death among persons who develop the combination of conditions. Segment in blue represents the period from age of onset to median age of death from the equivalent age and sex among the general population with and without comorbid conditions.

Figure 5 describes, for each diabetes-associated combination of comorbidities, the modeled median age of onset, the number of years lived with the MLTC combination and years lost. Many of the classic vascular–renal complications such as cerebrovascular disease, CHD, PVD, heart failure and CKD occurred later in life, with median onset in the mid-70s to early 80s, and were associated with 4–6 years of life lost. Combinations of diabetes with mental health conditions and learning disabilities, depression, alcohol dependence and asthma were characterized by an earlier median age of onset (late 50s to early 60s), of which all except alcohol dependence were associated with long periods of life spent (median 11–14 years) and all except asthma associated with considerably fewer (median 11–15 years) years of life. Chronic liver disease also had fairly early onset (before 70 years) and 12 years of life lost. A large set of discordant conditions, including rheumatoid arthritis, cancer, COPD and osteoarthritis, co-occurred among people with earlier deaths and thus fewer years spent with the combination of conditions. Additional conditions commonly associated with aging, including heart failure, atrial fibrillation and dementia, had later onset (late 70s to mid-80s of age) but fewer years spent with the condition (4–6 years).

Table 1 provides the contrast of the variation in differences in years lived and spent with different condition combinations from individual and community perspectives. When they occur for a given individual, combinations of diabetes with asthma, serious mental illness and depression have the highest number of years of life spent (15.4–16.6 years in women and 13.8–14.9 years in men), while combinations of diabetes with alcohol dependence and chronic liver disease were associated with the largest reduction in years of life from diagnosis, ranging from 12.5 to 15.7 years among men and women. Serious mental illness and learning disabilities, which had an earlier median onset, were associated with both a large number of years of life spent (14.0–15.4 years in women and 14.0–14.9 years in men) and lost (9.7–10.8 years in women and 10.3–10.6 years in men).

**Table 1:** Burden of diabetes-associated comorbid conditions, expressed in terms of years spent and reduction in years lived compared to those without diabetes, per individual with the condition (columns 1–2) and per community of 1,000 women and men

Per average individual with conditions		Per community of 1,000	
Women			
Years spent	Reduced years lived	Years spent	Reduced years lived
Asthma (16.6)	Alcohol dependence (15.7)	Hypertension (3,908)	Hypertension (1,255)
Depression (16.2)	Chronic liver disease (13.1)	Depression (2,261)	Depression (1,253)
Serious mental illness (15.4)	Learning disability (10.8)	Osteoarthritis (2,199)	Cancer (1,126)
Inflammatory bowel (14.4)	Epilepsy (10.7)	Asthma (1,681)	Osteoarthritis (821)
Learning disability (14.0)	Serious mental Illness (9.7)	CHD (1,280)	Cerebrovascular disease (788)
Hypertension (13.1)	Pulmonary heart disease (9.6)	Chronic pain (814)	Heart failure (786)
Chronic pain (12.2)	Cancer (9.3)	Cancer (813)	CHD (702)
Osteoarthritis (10.9)	Physical disability (9.2)	Osteoporosis (784)	COPD (689)
Rheumatoid arthritis (10.3)	Depression (9.0)	Cerebrovascular disease (674)	CKD (651)
Epilepsy (10.3)	Peripheral arterial disease (8.9)	Atrial fibrillation (612)	Asthma (635)
Men			
Years spent	Reduced years lived	Years spent	Reduced years lived
Serious mental illness (14.9)	Alcohol dependence (13.5)	Hypertension (4,318)	Hypertension (1,417)
Learning disability (14.0)	Chronic liver disease (12.5)	CHD (2,057)	Cancer (1,200)
Asthma (13.8)	Learning disability (10.6)	Osteoarthritis (1,617)	Heart failure (986)
Inflammatory bowel (13.3)	Serious mental Illness (10.3)	Depression (1,375)	Depression (963)
Depression (13.0)	Epilepsy (10.0)	Asthma (1,101)	CHD (900)
Hypertension (12.5)	Depression (9.0)	Atrial fibrillation (901)	Cerebrovascular disease (852)
CHD (10.6)	Physical disability (8.9)	Cancer (890)	Atrial fibrillation (835)
Osteoarthritis (9.7)	Pulmonary heart disease (8.8)	Cerebrovascular disease (742)	PVD (775)
Epilepsy (8.5)	Chronic pain (7.9)	PVD (653)	COPD (729)
Rheumatoid arthritis (8.4)	Severe interstitial lung disease (7.7)	COPD (650)	CKD (701)

When expressed in years spent with combinations of conditions at the community level (that is, per 1,000 population), the prevalence of conditions is considered. Diabetes with hypertension, depression, osteoarthritis, asthma and CHD account for the largest burden, ranging from 1,101 to 4,318 years per 1,000 population among both men and women. Depression, osteoarthritis, chronic pain and osteoporosis have larger roles in women, whereas CHD, atrial fibrillation, and PVD have a more prominent burden in men. In terms of years of life lost at the community level, hypertension, cancer, heart failure and depression comprise the leading four contributors in both men and women, accounting for a range of 788–1,417 years of life lost per 1,000 individuals. With the exception of depression, men lost more years of life associated with these conditions than women. The second leading effect of depression on years of life lost was in women, whereas a greater effect of hypertension and CHD on years of life lost was observed in men.

## Discussion

In this comprehensive examination of MLTCs in England, the morbidity associated with diabetes was extensive and diverse across the life course. We found that diabetes-related morbidity extends beyond the well-known classic complications. The patterns vary considerably by age and express their impact and burden in varied ways in terms of years spent or associated years of life lost. Diabetes-associated MLTCs in older age involve a broad spectrum of vascular conditions typically related to diabetes, including CHD, heart failure, CKD and stroke, but are also accompanied by the excess prevalence of discordant conditions, including osteoarthritis, cancers and asthma. By middle age, the absolute burden of MLTCs is already substantial, as over 30% of 50-year-olds with diabetes have at least three additional conditions, about 20 years younger for women and 15 years younger for men than the same number of MLTCs seen in the general population. Furthermore, this level of MLTCS was associated with about 12 fewer years of life. In young and middle-aged adults, however, depression, asthma and serious mental illness have more distinct and prominent roles. These observations are consistent with recent studies of MLTC clusters suggesting that morbidity emanating from diabetes and other cardiometabolic conditions develops along several axes, including those of vascular, mental health and musculoskeletal systems^28^.

Our study characterizes the burden of MLTCs in terms of years spent and years of life lost, depending on whether the perspective is individual or community burden. This reveals several distinct diabetes associations, including combinations with serious mental illness, alcohol dependence, chronic liver disease and learning disability that have very large impacts on both years spent and years lost when they occur for individuals affected, but given their low prevalence, have a modest impact at the community level. Cohort studies have established these associations and suggest they are multifactorial with genetic, environmental and treatment-related factors, but the degree to which they, in turn, predict more complex forms of MLTCs in subsequent life stages is unclear^29,30^. Some other comorbid conditions, such as asthma, exert a high burden in terms of years spent at both the individual and community level while having a modest association with years of life lost. The greatest burden on the community, in terms of years spent and years lost in people with diabetes, is seen for hypertension, depression, cancer and vascular conditions. The combination of osteoarthritis also carries a large burden on time spent with the condition, with a weaker association with years lost. Many of the common combinations may exist primarily through common etiological pathways or through a common risk factor, such as obesity^31^. Nevertheless, MLTC subtypes we observe are likely to have important influences not only on years of life lost but also on the quality of life of individuals and the demand and cost for health services.

Our findings are consistent with prior studies showing that diabetes is a central contributor to MLTCs in the general population and that the magnitude of association of diabetes with MLTCs is stronger in younger ages, particularly for more severe manifestations of MLTCs^5,16,18,32^. The strong association between diabetes and MLTCs could come from many processes. First, the classic macrovascular, microvascular, neuropathic and acute complications that accompany diabetes co-occur through common processes that serve as an engine for multicomponent MLTCs. For example, underlying drivers such as hyperglycemia, insulin resistance and inflammation may have driven a set of co-occurring conditions associated with diabetes, including cancers, infections, liver disease and chronic respiratory conditions. Although less specific to diabetes and of lower magnitude of association with diabetes than the traditional complications, these conditions appear to be increasing rather than decreasing and collectively affect large segments of the population with diabetes^5–7,33^. Second, rates of mortality among the population with diabetes have decreased precipitously over the past two decades, increasing the proportion of the population with diabetes in their eighth and ninth decades of life with longstanding diabetes^6,17^. The growing presence and/or awareness of nonspecific, noncardiovascular forms of diabetes-associated morbidity and mortality has been observed in several population studies over the past decade^7^. Third, the prevalence of obesity, particularly in young people both before and after diagnosis of diabetes, has increased dramatically and will also contribute to numerous pathways of MLTC development, resulting from chronic hyperglycemia, insulin resistance and inflammation that often occur in combination^34^. Fourth, social and environmental factors that exacerbate mental illness, dietary and behavioral risk factors and access to preventive care could all potentiate the development of more complex MLTCs. Finally, there may be underlying genetic or environmental drivers of MLTCs that simultaneously drive diabetes and MLTCs^35^.

The impact of MLTCs on potential quality of life and subsequent progression of morbidity, frailty and mortality is well established, with additional effects on caregivers. Health systems are challenged by the current burden of MLTCs because of the fragmented nature of many care systems and the need to coordinate diverse teams and specialties, as well as the increasing demands of polypharmacy and medication management, and behavioral and caregiver support. Whereas chronic care has emphasized specialized teams in many settings to address specific complications, the current findings indicate a need to consider the importance of generalist healthcare that can address the diversity of co-occurring conditions. Our findings highlight the substantial individual burden of having diabetes in combination with learning disability, depression, serious mental illness or epilepsy. This highlights the potential benefit of coordinating diabetes prevention and management alongside the primary specialty management of these conditions. People living with serious mental health conditions are at increased risk of developing diabetes. This may be a bidirectional association further influenced by the impact of some antipsychotic drugs on weight gain and diabetes risk^29^. Additionally, people with serious mental health conditions may have difficulty with diabetes self-management and attending appointments, making the coordination of mental and physical health services even more critical^36^. Our findings could inform efforts in medical education and training in the strategic integration of health service staff to support the challenges of diabetes-associated MLTCs. Accordingly, our data could help inform models of care to deliver more holistic approaches to chronic disease management for MLTCs.

The challenges of MLTCs expose at least three important gaps in the science of care and prevention. First, it is not clear whether the dominant risk factors of diabetes complications (such as hyperglycemia, insulin resistance, high blood pressure, hyperlipidemia, inflammation and obesity) also influenced MLTC progression at the same magnitude or whether different conduits need to be discovered and targeted. These cardinal drivers of classic diabetes complications may also variably affect nontraditional comorbid conditions, such as liver disease, respiratory disorders, infections, cancers, mental health and musculoskeletal problems^37^. Obesity, physical inactivity and social deprivation have each been identified as core contributors to MLTCs^27,28^^,37^^,38^. This underscores the need to understand how the spectrum and magnitude of risk factors for classic complications vary from other comorbid conditions and for the progression to MLTCs. Second, there has been limited examination of how models of care should best be changed or organized to adapt to growth in MLTCs. Research to date has focused on improving and adapting care team coordination, self-management support and medication management^39^. Third, it is unclear whether the best prevention approaches for MLTCs differ from the best approaches for their constituent conditions. Lifestyle behaviors, including physical activity, dietary interventions, weight loss and strength training, have received the most consideration, but the impact of interventions to reduce MLTCs remains unclear.

Several limitations in our analyses should be considered. Our data sources are derived predominantly from hospital and community-coded datasets (Supplementary Table 1) rather than GP datasets, so there may be under-ascertainment for those conditions usually diagnosed in GP. This means our estimates of time spent with conditions are likely to be conservative and systematically underestimated, particularly for some conditions typically diagnosed and managed in GP, such as combinations of diabetes with CKD (without end-stage renal failure), depression and hypertension. Moreover, diabetes-related eye diseases, which contribute to a substantial burden of disability among persons with diabetes, are not included in these analyses^40^. Our estimates of the age of median onset of diabetes-associated MLTCs are determined by the second condition and may obscure large variations in the onset of the first of the conditions. This variation may also have long-term effects on health and survival that may not be reflected in these estimates. Similarly, our analyses cannot determine the degree to which the differences we report across age strata are influenced by age itself or by inherent birth cohort effects. Our estimates of years of life lost with MLTC combinations do not adjust for additional conditions that may be differentially associated with the combinations; thus, the years of life lost should be interpreted as being associated with, but not necessarily caused by, the combination of conditions. The underlying data were not able to differentiate type 1 diabetes from type 2 diabetes. Given the relative number of cases of type 1 diabetes relative to type 2 diabetes (about 269,000 versus 3.5 million in England), this should have only a modest effect on the years of life lost estimate and negligible effect when the years of life lost are considered from an advanced age. Finally, we limited our consideration of MLTCs to a set of 35 prioritized conditions. This decision was in part due to the practical demands of modeling years of life spent and lost across more conditions, as combinations become rare^26^. Furthermore, this selection is in alignment with international consensus on the prioritization of conditions when considering public health approaches to address MLTCs^26^.

Despite these limitations, our study includes almost the entire population registered with a GP in England (over 98% of the population are registered with a GP), so the data are highly representative of the English population^25^. This large study reports data on diabetes-associated MLTCs in a whole national population of adults with diabetes and quantifies burden in terms of years spent and lost associated with MLTCs at individual and community level.

In summary, our analyses quantify the diabetes-associated burden of MLTCs and highlight the extensive burden and diverse forms it takes across the life course, taking both individual- and community-burden perspectives. In addition to supporting better health service resource allocation and commissioning decisions, these findings underscore the need for innovation and efforts to strengthen the agendas of both MLTC prevention and treatment.

## Methods

### Data sources and information governance

Our study population uses the National Bridges to Health Segmentation Dataset, which was developed and has been maintained since 2019 to support healthcare prioritization, planning and service evaluation for the NHS in England^27,41–43^. The dataset includes individuals registered with a GP in England since 2014, comprising 60,004,883 individuals. The segmentation dataset has been derived from more than 15 years of longitudinally accrued data from a number of national, predominantly secondary care, patient-level datasets in the National Commissioning Data Repository (NCDR)^42^, each of which was linked by a pseudonymized NHS number.

Data are collected and used in line with NHS England’s purposes as required under the statutory duties outlined in the NHS Act 2006 and Section 254 of the Health and Social Care Act 2012. Data are processed using best practice methodology underpinned by a data processing agreement between NHS England and Outcomes Based Healthcare (OBH), who produce the segmentation dataset on behalf of NHS England. This ensures controlled access by appropriate individuals to nonconsented, anonymized/pseudonymized data held on secure data environments entirely within the NHS England infrastructure. Data are processed for specific purposes only, including operational functions, service evaluation and service improvement. The current work supported these purposes, so ethics committee approval was not required. Where OBH has processed data, this has been agreed upon and is detailed in a Data Processing Agreement.

The present analyses are based on 46,748,714 adults aged 20 years and older who were alive as of 31 March 2019. We restricted data to the NHS financial year ending 2020 (that is, 1 April 2019 to 31 March 2020) to avoid distortion by the COVID-19 pandemic. The dataset includes information on sociodemographic data (such as age, sex (not gender), ethnicity and socioeconomic deprivation), geographical data (such as registered GP practice and mapped administrative NHS organization and location) and clinical diagnostic data, which are derived primarily from coded hospital records. Our analyses considered 35 long-term conditions, with the process of selection outlined previously^44^ and informed by a recent Delphi study that showed good concordance^26^. The inclusion of conditions beyond these 35 generates additional MLTC phenotypes of extremely low prevalence and lower priority for public health interventions. Furthermore, the computing intensity involved with modeling years spent and years lost due to condition combinations required an a priori prioritization of conditions. The 35 conditions were derived using data definitions based on logic and clinical codes (for example, International Classification of Diseases (ICD)-10 diagnostic codes, Office of Population Censuses and Surveys (OPCS) procedure codes and SNOMED CT codes) and were developed for each condition following extensive clinical review and evaluation^24^ (Supplementary Table 2).

The full list of source datasets used to derive the segmentation dataset, including the time over which data have been longitudinally accrued, is described in Supplementary Table 1. The National Diabetes Audit SNOMED codes and other condition definitions are available in online technical documents^24,45^. An antecedent validation study showed good concordance with established prevalence benchmarks, such as the England GP pay-for-performance scheme, called the Quality and Outcomes Framework, for the majority of conditions^24^.

### Statistical analysis

We calculated the point prevalence of all dual combinations of diabetes with other comorbid conditions, using the adult population in March 2020 as the denominator. We also calculated observed minus expected prevalence, where observed is the actual joint prevalence of diabetes with each condition and expected prevalence is the product of the diabetes prevalence in the general population and that of each condition, irrespective of diabetes status. Thus, expected prevalence refers to the joint prevalence of each duo that would be expected by chance with no etiologic association among the two conditions. We also calculated the number of comorbid conditions according to age and diabetes status.

To estimate the years spent and lost associated with types of diabetes-related MLTCs, we constructed a standard three-state illness-death Markov model^46,47^. The illness-death model (also known as the semi-competing risk model), used extensively to model time-to-event data, comprises the following three possible states: healthy, illness and death. The model allows the following three possible transitions: from healthy to illness, healthy to death or illness to death. Remission (from illness to healthy) is not permitted in this instance. The illness state is defined as the presence of the MLTC condition pair of interest, independent of the presence or absence of other conditions. The yearly likelihood of transition across states is assumed to be age-dependent, and rates are estimated through monthly observation of health status (long-term condition and mortality status) for all individuals from April 2019 to March 2020, as observed in the dataset. More precisely, the number of occurrences *n*_*i,j,a*_ of an individual of age *a* moving from state *i* to state *j* is tallied and probability distributed proportionally such that the likelihood, *P*_*i,j,a*_ of an individual age moving from state *i* to state *j* is given by ${P}_{i,\;j,a}=\frac{{n}_{i,\;j,a}}{\sum _{k\in S}{n}_{i,k,a}}$, where *S* is the set of possible final states. Where no transition data are available for a state at a given age, it is assumed the individuals remain in the same state as the time is incremented by 1 year. Because transition data are measured monthly and yearly data are required by the model, initially constructed monthly transition matrices *T*_*m*_ are exponentiated by a factor of 12 through matrix multiplication to convert to yearly transition matrices *T*_*y*_ according to the following equation:

$${{{T}}}_{{{y}}}={{{{T}}}_{{{m}}}}^{12}$$

For some combinations of conditions, the prevalence would be rare and insufficient data would be available to perform the calculations. To provide a sufficient distribution of ages moving into and out of the illness segment, analyses were restricted to those condition combinations where at least 1,000 observations were recorded of each transition type in the model. The model was limited to between 0 and 100 years. At this age, the vast majority of people have died, so extension beyond this age would have a negligible impact on model outputs.

Of the 35 long-term conditions, frailty was initially excluded from the analysis as remission was present in the data model but was not compatible with the form of the Markov model. There were insufficient transition observations for sickle cell disease, cystic fibrosis, autism, sarcoidosis and multiple sclerosis (as bimorbidity pairs with diabetes) to be included in the analysis.

The model was used to calculate the following five key metrics: lifetime risk of MLTC, median age at onset, years of life lived with (*Y*_LW_) the MLTC, age at death and years of life lost (*Y*_LL_) associated with the MLTCs. Lifetime risk, *L*_*r*_, is the probability that an individual at birth will enter the illness state at any point in their lifetime. This can be calculated by considering the proportion of the initial population that transitions from the healthy state to the illness state at a given age *a*, ${P}_{{\rm{healthy}}\to {\rm{ill}}}\left(a\right)$. This can be calculated by multiplying the proportion of the population who are in the healthy state at age *a*, *P*_healthy_ (*a*) by *P*_*i* = healthy*, j* *=* ill*, a*_, the probability of a healthy person entering the illness state in the immediate transition from age $a.$

$${P}_{{\rm{healthy}}\to {\rm{ill}}}(a)={P}_{{\rm{healthy}}}(a)\times {P}_{i\,=\,{\mathrm{healthy}},\,j\,=\,{\mathrm{ill}},\,a}$$

This can be summed over all ages in the model to give the total probability of transitioning to the illness state over a lifetime, the lifetime risk *L*_*r*_.

$${L}_{r}=\mathop{\sum }\limits_{a\,=\,0}^{100}{{{P}}}_{{\rm{healthy}}\to {\rm{ill}}}(a)$$

The years of life lost (at a given age ${a}$), ${Y}_{{\mathrm{LL}}}\left(a\right),$ is the difference in the survival function between those in the illness state and the survival function of a two-state alive-dead Markov model otherwise of the same form. An average measure of the years of life lost *Y*_LL_ experienced for the illness state of interest is calculated by a sum over all ages of ${Y}_{{\mathrm{LL}}}\left(a\right)$, weighted by the proportion of people entering the illness state who do so at that age.

$${Y}_{{\mathrm{LL}}}=\mathop{\sum }\limits_{a=0}^{100}\left(\,\frac{{{{P}}}_{{\rm{healthy}}\to {\rm{ill}}}(a)}{{L}_{r}}\times {Y}_{{\mathrm{LL}}}\left(a\right)\,\right)$$

The years lived with illness (given age $a$), *Y*_LW_ (*a*), is characterized by the survival function of the population who enters the illness state at that age. Again, an average measure, *Y*_LW_, is calculated using a weighted sum.

$${Y}_{{\mathrm{LW}}}=\mathop{\sum }\limits_{a=0}^{100}\left(\,\frac{{{{P}}}_{{\rm{healthy}}\to {\rm{ill}}}(a)}{{L}_{r}}\times {Y}_{{\mathrm{LW}}}\left(a\right)\,\right)$$

The median onset age of the illness state is extracted from the model by interpolating the age at the point where half of the total number of individuals that will transition into the illness state have transitioned, that is ${a}_{{\mathrm{median}}}$ is the integer value of *α* that minimizes $\left|\frac{{\sum }_{a=\propto }^{100}{P}_{{\mathrm{healthy}}\to {\mathrm{ill}}}\left(a\right)}{{L}_{r}}-0.5\right|$

These person-level metrics are conditional on individuals acquiring the ‘illnesses’ at some point in their lifetime. For the population as a whole, a community metric is defined as the total number of life years lost across 1,000 individuals, not all of which will enter the ‘illness’ state. Community metrics can be calculated by multiplying the above average metrics by the lifetime risk of the condition and scaling to 1,000 people.

$${C}_{{\mathrm{YLL}}}=1,000\times {Y}_{{\mathrm{LL}}}\times {L}_{r}$$

$${C}_{{\mathrm{YLW}}}=1,000\times {Y}_{{\mathrm{LW}}}\times {L}_{r}$$

### Reporting summary

Further information on research design is available in the Nature Portfolio Reporting Summary linked to this article.

## Online content

Any methods, additional references, Nature Portfolio reporting summaries, source data, extended data, supplementary information, acknowledgements, peer review information; details of author contributions and competing interests; and statements of data and code availability are available at 10.1038/s41591-024-03123-2.

## Supplementary information

Supplementary Information Supplementary Tables 1 and 2.

Reporting Summary

## Data Availability

As part of the support made available for local services and NHS, the Population and Person Insights dashboard, accessible by NHS organizations, presents aggregated data according to NHS administrative footprints, including at the national level (https://apps.model.nhs.uk/report/PaPi). The source data used in this evaluation and to derive the National Segmentation Dataset are from the NHS NCDR. The NCDR is a pseudonymized patient-level data repository managed by the NHS England (NHSE) Data Services team. It is used by NHS England for operational, service improvement and service evaluation purposes. It is designed to deliver consistent data processing, linkage and reporting services, mainly for the use of NHS England analytical teams to aid in the delivery of a wide range of projects, including the long-term plan. The data enable analysts to provide evidence in the form of reports and dashboards to support the NHS drive to improve health and well-being across England. Data Services was established by NHS England to ensure that information about the performance and impact of NHS services is available when decisions are made about the commissioning of health services. Data Services also ensures that information has been accessed legally in accordance with all applicable laws and mandatory guidance contained within the Health and Social Care Act 2012, the Care Act 2014, the Data Protection Act 2018 and the UK General Data Protection Regulation. The authors cannot provide direct access to the data, as this would circumvent NHS England’s research data access procedures. More information about the NCDR and how to contact NHS England Data Services is available at https://webarchive.nationalarchives.gov.uk/ukgwa/20231101051610/https://data.england.nhs.uk/ncdr/database/ (ref. ^42^).

## Code availability

The code is available in a GitHub repository at https://github.com/nhsengland/Markov-modelling-for-years-of-life-lost.

## References

[1] Gerstein, H. C. & Werstuck, G. H. Dysglycaemia, vasculopenia, and the chronic consequences of diabetes. *Lancet Diabetes Endocrinol.* **1**, 71–78 (2013). 24622269 10.1016/S2213-8587(13)70025-1

[2] Nathan, D. M. Long-term complications of diabetes mellitus. *N. Engl. J. Med.* **328**, 1676–1685 (1993). 8487827 10.1056/NEJM199306103282306

[3] Gregg, E. W. et al. Changes in diabetes-related complications in the United States, 1990–2010. *N. Engl. J. Med.* **370**, 1514–1523 (2014). 24738668 10.1056/NEJMoa1310799

[4] Harding, J. L., Pavkov, M. E., Magliano, D. J., Shaw, J. E. & Gregg, E. W. Global trends in diabetes complications: a review of current evidence. *Diabetologia* **62**, 3–16 (2019). 30171279 10.1007/s00125-018-4711-2

[5] Pearson-Stuttard, J. et al. Trends in leading causes of hospitalisation of adults with diabetes in England from 2003 to 2018: an epidemiological analysis of linked primary care records. *Lancet Diabetes Endocrinol.* **10**, 46–57 (2022). 34861153 10.1016/S2213-8587(21)00288-6 PMC8672063

[6] Gregg, E. W. et al. Trends in cause-specific mortality among adults with and without diagnosed diabetes in the USA: an epidemiological analysis of linked national survey and vital statistics data. *Lancet* **391**, 2430–2440 (2018). 29784146 10.1016/S0140-6736(18)30314-3

[7] Tomic, D., Salim, A., Morton, J. I., Magliano, D. J. & Shaw, J. E. Reasons for hospitalisation in Australians with type 2 diabetes compared to the general population, 2010–2017. *Diabetes Res. Clin. Pract.* **194**, 110143 (2022). 36370894 10.1016/j.diabres.2022.110143

[8] Gregg, E. W., Sattar, N. & Ali, M. K. The changing face of diabetes complications. *Lancet Diabetes Endocrinol.* **4**, 537–547 (2016). 27156051 10.1016/S2213-8587(16)30010-9

[9] Koyama, A. K. et al. Trends in lifetime risk and years of potential life lost from diabetes in the United States, 1997–2018. *PLoS ONE* **17**, e0268805 (2022). 35609056 10.1371/journal.pone.0268805 PMC9129010

[10] Lingvay, I., Sumithran, P., Cohen, R. V. & le Roux, C. W. Obesity management as a primary treatment goal for type 2 diabetes: time to reframe the conversation. *Lancet* **399**, 394–405 (2022). 34600604 10.1016/S0140-6736(21)01919-X

[11] Kahn, S. E., Cooper, M. E. & Del Prato, S. Pathophysiology and treatment of type 2 diabetes: perspectives on the past, present, and future. *Lancet* **383**, 1068–1083 (2014). 24315620 10.1016/S0140-6736(13)62154-6 PMC4226760

[12] Dibato, J. E. et al. Association of cardiometabolic multimorbidity and depression with cardiovascular events in early-onset adult type 2 diabetes: a multiethnic study in the U.S. *Diabetes Care* **44**, 231–239 (2021). 33177170 10.2337/dc20-2045

[13] Barnett, K. et al. Epidemiology of multimorbidity and implications for health care, research, and medical education: a cross-sectional study. *Lancet* **380**, 37–43 (2012). 22579043 10.1016/S0140-6736(12)60240-2

[14] Whitty, C. J. M. et al. Rising to the challenge of multimorbidity. *BMJ* **368**, l6964 (2020). 31907164 10.1136/bmj.l6964 PMC7190283

[15] Tran, J. et al. Patterns and temporal trends of comorbidity among adult patients with incident cardiovascular disease in the UK between 2000 and 2014: a population-based cohort study. *PLoS Med.* **15**, e1002513 (2018). 29509757 10.1371/journal.pmed.1002513 PMC5839540

[16] Cicek, M., Buckley, J., Pearson-Stuttard, J. & Gregg, E. W. Characterizing multimorbidity from type 2 diabetes: insights from clustering approaches. *Endocrinol. Metab. Clin. North Am.* **50**, 531–558 (2021). 34399960 10.1016/j.ecl.2021.05.012 PMC8383848

[17] Khunti, K. et al. Diabetes and multiple long-term conditions: a review of our current global health challenge. *Diabetes Care* **46**, 2092–2101 (2023). 38011523 10.2337/dci23-0035 PMC10698221

[18] Nowakowska, M. et al. The comorbidity burden of type 2 diabetes mellitus: patterns, clusters and predictions from a large English primary care cohort. *BMC Med.* **17**, 145 (2019). 31345214 10.1186/s12916-019-1373-y PMC6659216

[19] Stirland, L. E. et al. Measuring multimorbidity beyond counting diseases: systematic review of community and population studies and guide to index choice. *BMJ* **368**, m160 (2020). 32071114 10.1136/bmj.m160 PMC7190061

[20] Bisquera, A. et al. Identifying longitudinal clusters of multimorbidity in an urban setting: a population-based cross-sectional study. *Lancet Reg. Health Eur.* **3**, 100047 (2021). 34557797 10.1016/j.lanepe.2021.100047 PMC8454750

[21] Aga, F., Dunbar, S. B., Kebede, T. & Gary, R. A. The role of concordant and discordant comorbidities on performance of self-care behaviors in adults with type 2 diabetes: a systematic review. *Diabetes Metab. Syndr. Obes.* **12**, 333–356 (2019). 31114271 10.2147/DMSO.S186758 PMC6497834

[22] Narayan, K. M., Boyle, J. P., Thompson, T. J., Sorensen, S. W. & Williamson, D. F. Lifetime risk for diabetes mellitus in the United States. *JAMA* **290**, 1884–1890 (2003). 14532317 10.1001/jama.290.14.1884

[23] NHSE subsegment/condition definitions. *Outcomes Based Healthcare* https://outcomesbasedhealthcare.com/condition-definitions-overview/ (2018).

[24] NHS England segmentation dataset reference guide—comparison against QOF. *Outcomes Based Healthcare* https://outcomesbasedhealthcare.com/nhse-segmentation-dataset-reference-guide/#comparison-to-QOF (2024).

[25] Herrett, E. et al. Data resource profile: Clinical Practice Research Datalink (CPRD). *Int J. Epidemiol.* **44**, 827–836 (2015). 26050254 10.1093/ije/dyv098 PMC4521131

[26] Ho, I. S. et al. Variation in the estimated prevalence of multimorbidity: systematic review and meta-analysis of 193 international studies. *BMJ Open* **12**, e057017 (2022). 35487738 10.1136/bmjopen-2021-057017 PMC9058768

[27] Valabhji, J. et al. Prevalence of multiple long-term conditions (multimorbidity) in England: a whole population study of over 60 million people. *J. R. Soc. Med.* **117**, 104–117 (2023). 37905525 10.1177/01410768231206033 PMC11046366

[28] Aguado, A., Moratalla-Navarro, F., López-Simarro F. & Moreno, V. MorbiNet: multimorbidity networks in adult general population. Analysis of type 2 diabetes mellitus comorbidity. *Sci. Rep.*10.1038/s41598-020-59336-1 (2020). 10.1038/s41598-020-59336-1 PMC7016191 32051506

[29] Holt, R. I. & Mitchell, A. J. Diabetes mellitus and severe mental illness: mechanisms and clinical implications. *Nat. Rev. Endocrinol.* **11**, 79–89 (2015). 25445848 10.1038/nrendo.2014.203

[30] Mezuk, B., Eaton, W. W., Albrecht, S. & Golden, S. H. Depression and type 2 diabetes over the lifespan: a meta-analysis. *Diabetes Care* **31**, 2383–2390 (2008). 19033418 10.2337/dc08-0985 PMC2584200

[31] Khunti, K. F. et al. Weight change and risk of obesity-related complications: a retrospective population-based cohort study of a UK primary care database. *Diabetes Obes. Metab.* **25**, 2669–2679 (2023). 37283064 10.1111/dom.15154

[32] Zghebi, S. S., Steinke, D. T., Rutter, M. K. & Ashcroft, D. M. Eleven-year multimorbidity burden among 637 255 people with and without type 2 diabetes: a population-based study using primary care and linked hospitalisation data. *BMJ Open* **10**, e033866 (2020). 32611677 10.1136/bmjopen-2019-033866 PMC7358107

[33] Tomic, D., Shaw, J. E. & Magliano, D. J. The burden and risks of emerging complications of diabetes mellitus. *Nat. Rev. Endocrinol.* **18**, 525–539 (2022). 35668219 10.1038/s41574-022-00690-7 PMC9169030

[34] Sattar, N., McMurray, J. J. V., McInnes, I. B., Aroda, V. R. & Lean, M. E. J. Treating chronic diseases without tackling excess adiposity promotes multimorbidity. *Lancet Diabetes Endocrinol.* **11**, 58–62 (2023). 36460014 10.1016/S2213-8587(22)00317-5

[35] Langenberg, C., Hingorani, A. D. & Whitty, C. J. M. Biological and functional multimorbidity-from mechanisms to management. *Nat. Med.* **29**, 1649–1657 (2023). 37464031 10.1038/s41591-023-02420-6

[36] Bellass, S. et al. Living with diabetes alongside a severe mental illness: a qualitative exploration with people with severe mental illness, family members and healthcare staff. *Diabet. Med.* **38**, e14562 (2021). 33772867 10.1111/dme.14562

[37] Kivimäki, M. et al. Body-mass index and risk of obesity-related complex multimorbidity: an observational multicohort study. *Lancet Diabetes Endocrinol.* **10**, 253–263 (2022). 35248171 10.1016/S2213-8587(22)00033-X PMC8938400

[38] Dhalwani, N. N. et al. Association between lifestyle factors and the incidence of multimorbidity in an older English population. *J. Gerontol. A Biol. Sci. Med. Sci.* **72**, 528–534 (2017). 27470302 10.1093/gerona/glw146

[39] Smith, S. M., Wallace, E., Clyne, B., Boland, F. & Fortin, M. Interventions for improving outcomes in patients with multimorbidity in primary care and community setting: a systematic review. *Syst. Rev.* **10**, 271 (2021). 34666828 10.1186/s13643-021-01817-z PMC8527775

[40] Burton, M. J. et al. The Lancet Global Health Commission on global eye health: vision beyond 2020. *Lancet Glob. Health* **9**, e489–e551 (2021). 33607016 10.1016/S2214-109X(20)30488-5 PMC7966694

[41] Barron, E. et al. Associations of type 1 and type 2 diabetes with COVID-19-related mortality in England: a whole-population study. *Lancet Diabetes Endocrinol.* **8**, 813–822 (2020). 32798472 10.1016/S2213-8587(20)30272-2 PMC7426088

[42] NHS England. NCDR Reference Library. *UK Government Web Archive* https://webarchive.nationalarchives.gov.uk/ukgwa/20231101051610/https://data.england.nhs.uk/ncdr/database/ (archived 1 November 2023).

[43] Lynn, J., Straube, B. M., Bell, K. M., Jencks, S. F. & Kambic, R. T. Using population segmentation to provide better health care for all: the ‘Bridges to Health’ model. *Milbank Q.* **85**, 185–208 (2007). 17517112 10.1111/j.1468-0009.2007.00483.x PMC2690331

[44] Hafezparast, N. et al. Adapting the definition of multimorbidity—development of a locality-based consensus for selecting included long term conditions. *BMC Fam. Pract.* **22**, 124 (2021). 34162331 10.1186/s12875-021-01477-x PMC8223362

[45] Enhanced services (ES), vaccination and immunisation (V&I) and core contract components (CC) business rules 2023–2024. *NHS Digital* https://digital.nhs.uk/data-and-information/data-collections-and-data-sets/data-collections/quality-and-outcomes-framework-qof/quality-and-outcome-framework-qof-business-rules/enhanced-services-es-vaccination-and-immunisation-vi-and-core-contract-components-cc-business-rules-2023-2024 (2023).

[46] Meira-Machado, L., de Uña-Alvarez, J., Cadarso-Suárez, C. & Andersen, P. K. Multi-state models for the analysis of time-to-event data. *Stat. Methods Med. Res.* **18**, 195–222 (2009). 18562394 10.1177/0962280208092301 PMC2692556

[47] Sonnenberg, F. A. & Beck, J. R. Markov models in medical decision making: a practical guide. *Med. Decis. Making* **13**, 322–338 (1993). 8246705 10.1177/0272989X9301300409

