# Supplementary material for: The burden of diabetes-associated multiple long-term conditions on years of life spent and lost
Source: Nat Med. 2024 Aug 1;30(10):2830–7. doi: 10.1038/s41591-024-03123-2 (PMC11485235; doi:10.1038/s41591-024-03123-2)
Supplement: Supplementary file 1 — Supplementary Tables 1 and 2. [file 41591_2024_3123_MOESM1_ESM.pdf]

nature medicine

Supplementary information https://doi.org/10.1038/s41591-024-03123-2

The burden of diabetes-associated multiple long-term conditions on years of life spent and lost

In the format provided by the authors and unedited

Table S1. Source datasets used to derive the National Bridges to Health Segmentation Dataset, and the time over which data has been longitudinally accrued for each.

Dataset	Dataset start date	Dataset end date*
Master Patient Index (MPI)	Aug-14	Aug-21
SUS Admitted Patient Care (APC)	Apr-08	Aug-21
SUS Outpatient (OP)	Apr-08	Aug-21
SUS Accident & Emergency (A&E)	Apr-08	Mar-20
SUS Emergency Care Dataset (ECDS)	Oct-17	Aug-21
Hospital Frailty Risk Score (derived from SUS APC)	Apr-14	Aug-21
Community Services Dataset (CSDS)	Oct-17	Aug-21
Assuring Transformation (AT)	Feb-15	Aug-21
Service Level Agreement Monitoring (SLAM) for specialised or tertiary services	Apr-16	Aug-21
Mental Health Minimum Data Set (MHMDS)	Apr-13	Mar-14
Mental Health Learning Disabilities Data Set (MHLDDS)	Apr-14	Dec-15
Mental Health Services Data Set (MHSDS)	Apr-16	Aug-21
Improving Access to Psychological Therapies (IAPT)	Apr-13	Aug-21
Maternity Services Data Set (MSDS)	Apr-19	Aug-21
National Diabetes Audit (NDA)	Jan-14	Aug-21

* Only data up to the end of March 2020 have been used to calculate the prevalence of multiple long-term conditions in England on 31st March 2020

Table S2. The thirty-five conditions included in the National Bridges to Health Segmentation Dataset mapped against the conditions suggested by the recent Delphi study.

Bridges to Health Segmentation Dataset Condition List Ordered as list of LTCs (with severity of certain conditions grouped/paired)	*Delphi Condition List*	
Alcohol Dependence	*Usually include*	*Drug and alcohol misuse*
Asthma	*Always include*	*Asthma*
Atrial Fibrillation	*Usually include*	*Arrhythmia*
Autism	*Usually include*	*Autism*
Bronchiectasis	*Usually include*	*Bronchiectasis*
Cancer & Incurable Cancer	*Always include*	*Metastatic cancer, haematological cancers, solid organ cancers, melanoma (usually include), treated cancer requiring surveillance (usually include)*
Cerebrovascular Disease	*Always include*	*Stroke, TIA (usually include)*
Chronic Kidney Disease & End Stage Renal Failure	*Always include*	*Chronic Kidney Disease & End Stage Renal Failure*
Chronic Liver Disease & Liver Failure	*Always include*	*Chronic Liver Disease*
Chronic Pain	*Usually include*	*Peripheral neuropathy*
COPD & Severe COPD	*Always include*	*COPD*
Coronary Heart Disease	*Always include*	*Coronary Artery Disease*
Cystic Fibrosis	*Always include*	*Cystic Fibrosis*
Dementia	*Always include*	*Dementia*
Depression	*Usually include*	
Diabetes	*Always include*	*Diabetes*
Epilepsy	*Always include*	*Epilepsy*
Heart Failure & Severe Heart Failure	*Always include*	*Heart Failure*
Hypertension	*Usually include*	*Treated or untreated hypertension*
Inflammatory Bowel Disease	*Always include*	*Inflammatory Bowel Disease*
Intermediate & High Frailty Risk (HFRS)		
Learning Disability		
Multiple Sclerosis	*Always include*	*Multiple Sclerosis*
Neurological Organ Failure		
Osteoarthritis	*Usually include*	*Osteoarthritis*
Osteoporosis	*Usually include*	*Osteoporosis*
Parkinson's Disease	*Always include*	*Parkinson's Disease*
Peripheral Vascular Disease	*Always include*	*Peripheral Arterial Disease*
Physical Disability	*Usually include*	*Vision or hearing impairment that cannot be corrected, long term MSK problems due to injury, congenital disease and chromosomal abnormalities, paralysis (other than stroke)*
Pulmonary Heart Disease		
Rheumatoid Arthritis	*Always include*	*Connective Tissue Disorders*
Sarcoidosis		

Bridges to Health Segmentation Dataset Condition List<br>*Ordered as list of LTCs (with severity of certain conditions grouped/paired)*	*Delphi Condition List*	
Serious Mental Illness	*Always include*	*Schizophrenia / Bipolar (usually include)*
Severe Interstitial Lung Disease		
Sickle Cell Disease		
Not included	Always include	HIV/AIDs
Not included	Usually include	Heart valve disorders, pancreatic disease, thyroid disorders, anaemia, chronic Lyme disease, eating disorders, tuberculosis, endometriosis, peptic ulcer, Post Traumatic Stress Disorder, post-acute Covid-19, benign cerebral tumours, chronic urinary tract infection, Meniere's disease, anxiety, gout
